# Sudan ebolavirus long recovered survivors produce GP-specific Abs that are of the IgG1 subclass and preferentially bind FcγRI

**DOI:** 10.1038/s41598-017-06226-8

**Published:** 2017-07-20

**Authors:** Olga Radinsky, Avishay Edri, Michael Brusilovsky, Shlomit Fedida-Metula, Ariel Sobarzo, Orly Gershoni-Yahalom, Julius Lutwama, John Dye, Leslie Lobel, Angel Porgador

**Affiliations:** 1 The Shraga Segal Department of Microbiology, Immunology and Genetics, Faculty of Health Sciences, Ben-Gurion University of the Negev, Beer Sheva, Israel; 2 National Institute for Biotechnology in the Negev, Ben-Gurion University of the Negev, Beer Sheva, Israel; 3 Virology Division – U.S. Army Medical Research Institute of Infectious Diseases 1425 Porter St., Fort Detrick, Frederick, Maryland 21701 USA; 4 Department of Arbovirology, Emerging and Re-emerging Infection, Uganda Virus Research Institute, Entebbe, Uganda

## Abstract

Ebolavirus is a highly lethal pathogen, causing a severe hemorrhagic disease with a high fatality rate. To better understand immune correlates of protection by virus specific IgG, we investigated the evolution of the Fcγ receptors (FcγRs)-activating capabilities of antiviral IgG in serum samples of long recovered survivors. To this end, longitudinal serum samples from survivors of Sudan ebolavirus (SUDV) infection, studied over years, were examined for the presence of Ebola-GP specific IgG subclasses, and for their binding to FcγRs. We developed a cell-based reporter system to quantitate pathogen-specific antibody binding to FcγRIIIA, FcγRIIA, FcγRIIB and FcγRI. With this system, we demonstrate that anti-GP-specific stimulation of the FcγRI reporter by survivors’ sera was substantially high one year after acute infection, with a slight reduction in activity over a decade post infection. We further demonstrate that GP-specific IgG1 is by far the seroprevalent subclass that retained and even enhanced its presence in the sera, over ten years post infection; the prevalence of other GP-specific IgG subclasses was considerably reduced over time. In accordance, GP-specific FcγRI reporter response and GP-specific total IgG1 subclass correlated in the studied group of Ebola survivors. These observations are important for further informing Ebola vaccine and therapeutic development.

## Introduction

Ebolavirus hemorrhagic fever (EHF) is a severe disease, caused by a members of the filoviridae family, with an as yet undefined reservoir and a high case fatality rate^1^. Recent outbreaks in West Africa have demonstrated the significant human and societal burden of outbreaks of this virus^2, 3^. Defining a comprehensive profile of the native humoral and cellular immune responses, which correlate with protective immune responses, is key for effective countermeasure development. Studies that examined the pathogenesis of ebolavirus infection in humans indicate that recovery is largely dependent upon, and associated with, the development of both cell-mediated and humoral immune responses^4–6^. Previous studies that examined survivors and asymptomatic cases demonstrated the presence of significant levels of virus-specific IgM and IgG associated with a temporary, early and strong inflammatory response^7–9^. In addition, recent evidence from long recovered SUDV survivors has demonstrated several distinctive profiles of immunity, which included persistent and strong IgG neutralizing humoral immunity more than a decade post infection in some survivors^10, 11^. However, other studies have also documented a significant number of convalesced patients with no residual humoral or cell mediated memory immune responses^12^. As such, it is clear that a comprehensive picture of immunity to ebolavirus is lacking, as well as an understanding of the interplay between components of the human immune system. To shed greater light on immune factors that correlate with survival, we describe herein a novel study of immune responses in Sudan ebolavirus survivors, which suggest a coordinated response between the humoral recognition and activation components of immunity in ebolavirus hemorrhagic fever (EHF).

Human Fcγ receptors (FcγRs) are a family of proteins that bind specifically to the Fc region of IgGs eliciting various immunological responses^13^. Measuring the FcγR-activating capabilities of antiviral IgG augments definition of immune correlates of protection against infections and/or infection-induced disease progression. Three different types of Fcγ receptors are displayed on the cell surface of human leukocytes: FcγRI (CD64), FcγRII (types A, B, and C, collectively known as CD32), and FcγRIII (types A and B, collectively known as CD16)^14^. Binding affinity of human IgG Fc to a corresponding FcγR is dictated by both the IgG-Fc subclass (IgG1, IgG2, IgG3 and IgG4) and changes in a single N-linked glycan located in the CH2 domain of the IgG Fc^15–18^. For example, IgG1 is considered as the subclass with the highest affinity to FcγRs^19–21^; yet, fucose, galactose and sialic acid modifications decrease or increase its affinity to FcγRIII and FcγRII^22^.

Destruction of IgG-coated targets by cell-mediated pathways begins with an interaction between the IgG Fc region and FcγRs on the surface of leukocytes. As such, several studies analyzed binding of pathogen-specific antibodies to FcγRs^23–26^. Mahan *et al*. demonstrated that dramatic differences exist in bulk IgG glycosylation among individuals in distinct geographical locations, however, HIV-specific immunization is able to overcome these differences and elicit antigen-specific antibodies with similar antibody glycosylation^26^. Their data strongly suggest that the immune system naturally drives antibody glycosylation in an antigen-specific manner. We aimed to further study FcγRs’ binding of pathogen-specific Abs by developing a cell-based reporter system to quantitate antibody binding to FcγRIII, FcγRII and FcγRI. We then investigated sera from SUDV survivors for the SUDV glycoprotein-specific Ab response. Long recovered survivors of SUDV infection, with no additional clinical reported exposures, enables assessment of long-term B-cell memory to an isolated single infection. We observed that IgG1 is the dominant GP-specific IgG subclass that significantly remains detectable more than a decade post infection. Interestingly, it correlates with prominent binding to FcγRI, as compared to binding to FcγRIIIA, FcγRIIA and FcγRIIB.

## Results

### Development of four FcγRs reporter system

**Figure 1:**
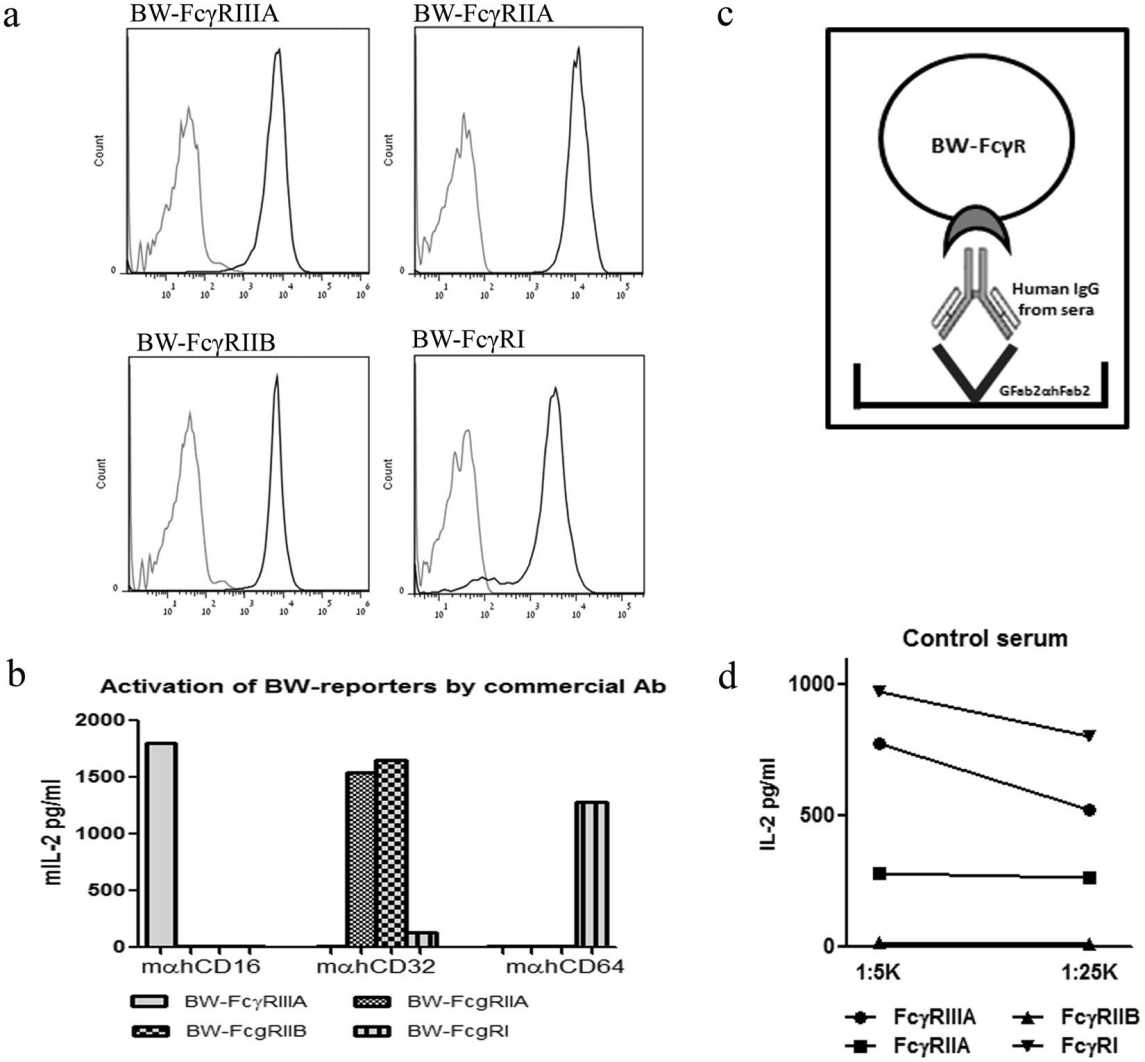
Production and activation of the BW-FcγR reporter cells by IgG. (**a**) Detection of stable transfected Fcγ Receptors was screened by flow cytometry using combined staining with anti-human CD-16/ 32/ 64 -black line, secondary APC- grey line. (**b**) Activation of BW reporter cells by commercial mAb. 96-well culture plates were pre-coated with Goat anti-mouse IgG (2 μg/ml), blocked, washed and coated with mAbs specific for each FcγR 250 ng/ml. Following additional washing, BW-FcγR cell lines were added, 50*10^3^ cell/well. mIL-2 secretion was determined by ELISA kit after 16 h incubation. (**c**) IgG mediated activation process diagram of BW-FcγR reporter cells. Coating of plates with goat Fab2 portion anti human Fab2 of IgG, adding human sera containing IgG followed by seeding with reporter cells 50*10^3^ cells/well. mIL-2 secretion of activated cells determined by ELISA after 16 h incubation. (**d**) Activation of BW reporter cells by control sera (1:5 K and 1:25 K dilution). mIL-2 secretion was determined by ELISA after 16 h incubation.

Murine BW5147 thymoma cells, transfected with chimeras composed of CD3ζ chain fused to the extracellular part of a certain surface receptor, can be stimulated to secrete endogenous murine IL-2 following activation of the fused surface receptor^27^. Several studies employed these cells to study various chimeric receptors, particularly human cell surface receptors, taking advantage of the lack of endogenous TCR and ζ as well as the studied receptor in BW5147 cells^28, 29^. We developed four BW5147-based human FcγR reporters based on transfection of chimeric CD3ζ-FcγRIIIA (CD16A), CD3ζ-FcγRIIA (CD32A), CD3ζ-FcγRIIB (CD32B) and CD3ζ-FcγRI (CD64). Since BW5147 lacks expression of endogenous murine Fcγ receptors, we did not expect cross-reactivity derived from the function of endogenous murine FcγRs^30^. Figure 1a shows the homogenous high level expression of the transfected chimeric human FcγRs in the selected BW5147 clones. To verify that binding to the transfected receptors activates the different FcγR reporters to secrete murine IL-2, we incubated the cells in wells coated with commercial mAbs recognizing the specific receptors (clone 3G8 anti-human FcγRIIIA, clone FUN-2 anti hFcγRIIA and anti h FcγRIIB, & clone 10.1 anti hFcγRI). Substantial levels of murine IL-2 were secreted by the activated reporters (Fig. 1b). We then aimed to assess the response of the four reporters to the Fc of IgGs in human sera employing the protocol depicted in Fig. 1c. 96-well plates were pre-coated with commercial goat(Fab)_2_-anti-human IgG(Fab)_2_, then blocked and coated with titrated amounts of human sera, followed by addition of reporter cells. IL-2 secreted into the supernatant represents binding of the transfected Fcγ receptors by the upward-pointing Fc of the sera IgGs interacting with the plastic-coated anti human IgG(Fab)_2_. IgG1 constitutes more than 70% of the IgGs in normal human sera, and IgG1-Fc affinity to hFcγRI is the highest, followed by its affinity to hFcγRIIIA^19, 21, 31^. In accordance, when tested on a control human serum, FcγRI reporter manifested the highest levels of secreted IL-2 followed by the FcγRIIIA reporter (Fig. 1d).

### FcγRs’ binding potency of long recovered SUDV survivors is similar to non-infected controls

**Figure 2:**
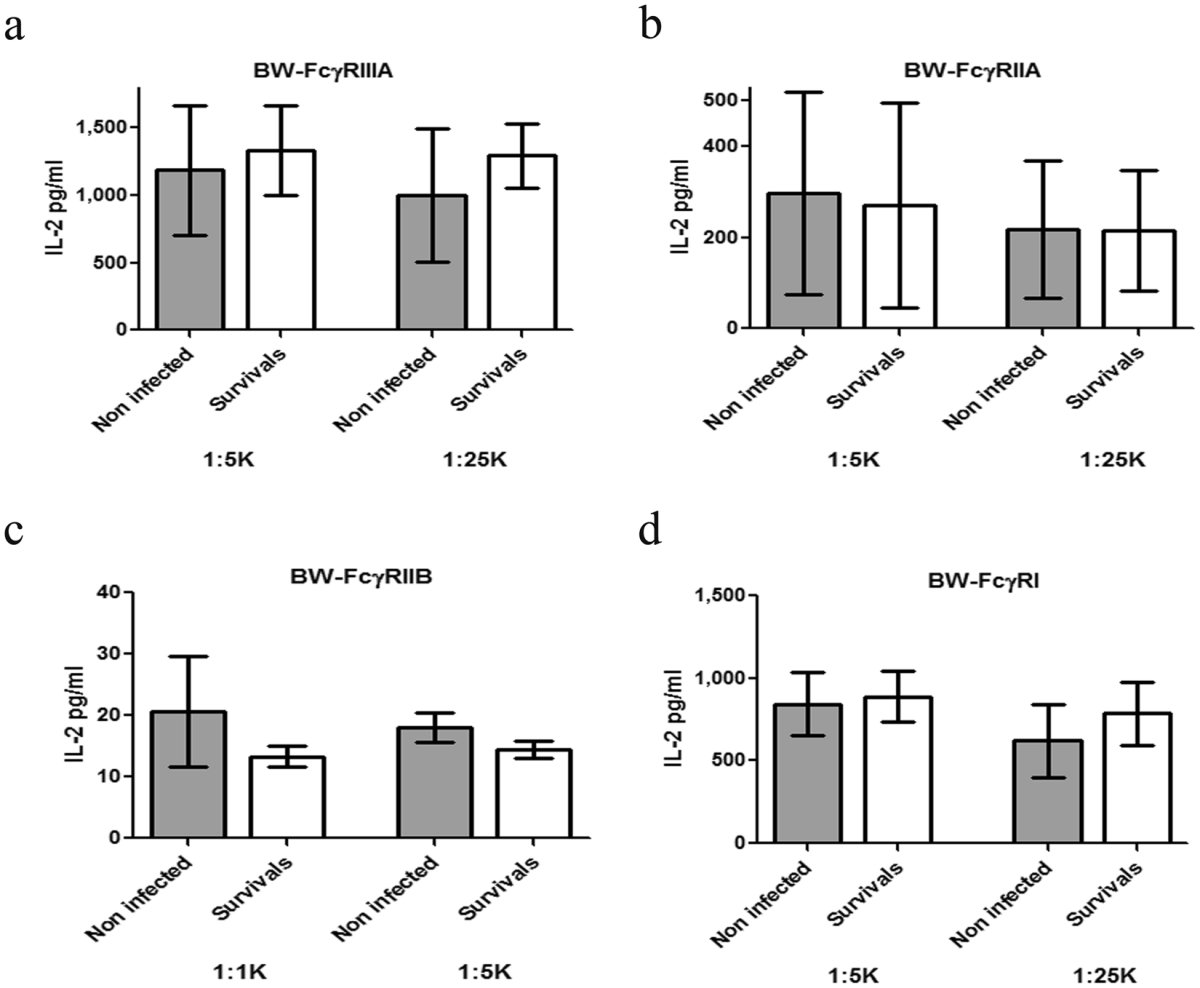
Binding and activation of the reporters by sera from survivors of SUDV outbreak. Total IgG dependent activation through FcγRs with goat- anti human IgG. After pre-coating with goat-anti human IgG (2 µg/ml) the activation assay was performed (as described in the legend of Fig. 1) using a total of 14 non-infected and 14 survivors’ serum samples (including 5 serum samples from a longitudinal study for the GP-Gulu specificity). 1:1 K and 1:5 K sera dilution was used for BW-FcγRIIB response, and 1:5 K and 1:25 K for BW-FcγRIIIA/IIA/I response. mIL-2 secretion was determined by ELISA kit after 16 h incubation.

Following testing of the FcγR reporters, we further investigated binding to the reporters of sera from human survivors of SUDV hemorrhagic fever. For controls, sera was collected from unexposed individuals who were from the same districts where the outbreak occurred but were never diagnosed with Ebola virus infection and/or were negative by PCR for SUDV^12^. We compared sera from 14 non-infected and 14 ebolavirus decade long survivors (Fig. 2). We chose to compare sera from long recovered survivors to non-infected individuals to avoid the short-term systemic effect of the Ebola disease on circulating IgG. Sera was tested in two dilutions (1:5 K and 1:25 K) except for the FcγRIIB reporter (1:1 K and 1:5 K). Overall, substantial IL-2 secretion was recorded for FcγRIIIA and FcγRI reporters in both non-infected and survivor groups. IL-2 secretion recorded for the FcγRIIA reporter was nearly 3-fold lower in both groups, whereas IL-2 secretion recorded for the FcγRIIB was significantly lower as compared to the other 3 reporters (Fig. 2). To summarize, reporter-binding profiles of circulating IgG from survivors and non-infected groups did not manifest differences that could point to a specific advantage/characteristic of the survivor group.

### FcγRI response is consistently prominent in GP-specific Ab response in long recovered SUDV survivors

**Figure 3:**
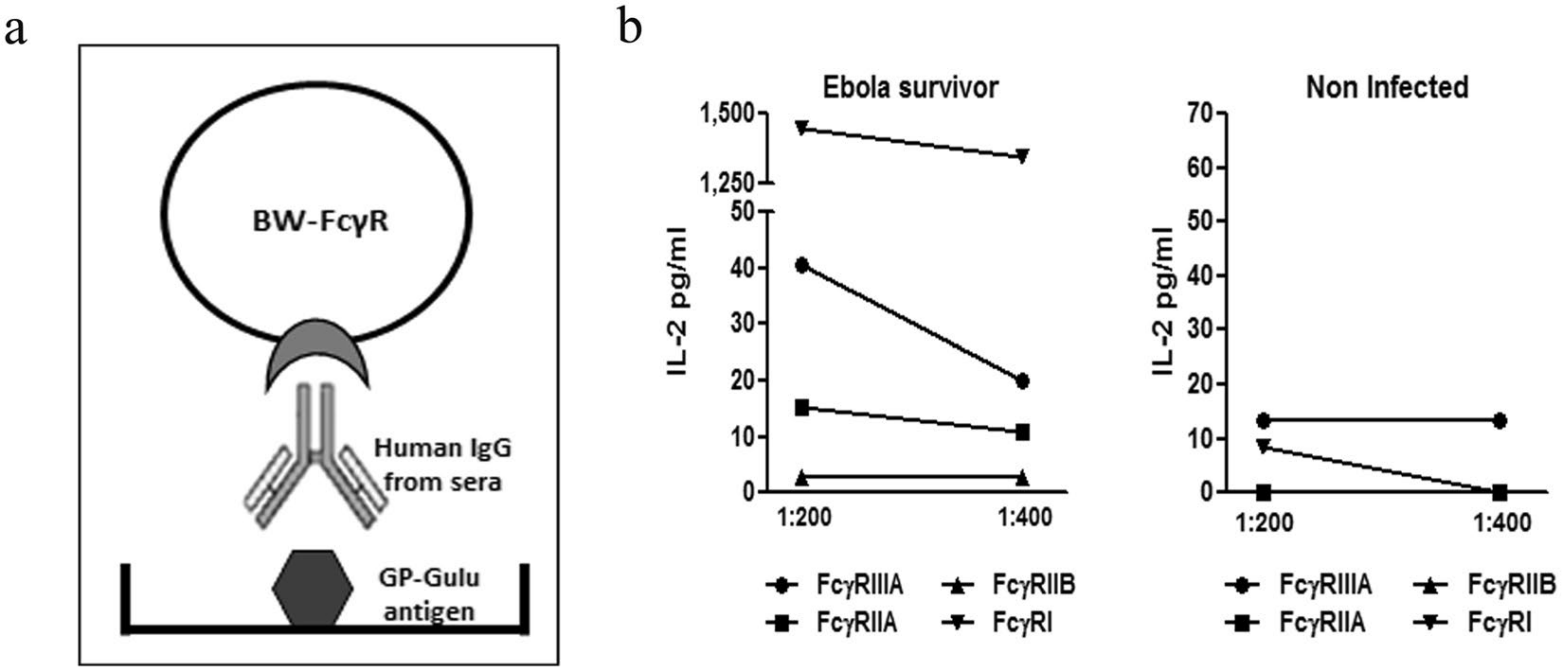
Binding of Ebola GP-specific antibodies found in survivors sera to FcγR reporters. Serum samples from non-infected controls was compared to SUDV survivors by its BW-FcγR activation capacity. (**a**) Diagram of antigen specific IgG mediated activation of BW-FcγR cells. Coating of plates with GP-Gulu antigen (5 µg/ml), addition of human sera following seeding with reporter cells at 50*10^3^ cells/well. mIL-2 secretion of activated cell determined by ELISA after 16 h incubation. (**b**) Serum samples from SUDV survivor and non-infected patient diluted 1:200 and 1:400 were added to pre-coated plates with GP-Gulu antigen (5 µg/ml). After activation assay, mIL-2 secretion was determined by ELISA after 16 h.

We therefore further assessed the binding to FcγR reporters of Ebola GP-Gulu specific antibodies found in survivors’ sera. We employed the protocol depicted in Fig. 3a. 96-well plates were pre-coated with recombinant GP-Gulu antigen, then blocked and coated with titrated amounts of sera, followed by addition of reporter cells. IL-2 secreted into the supernatant represented the binding of the transfected Fcγ receptors by the upward-pointing Fc of the IgGs interacting with the plastic-coated GP-Gulu antigen. We again compared sera from non-infected and ebolavirus survivors; sera were tested at two dilutions (1:200 and 1:400). Specificity of the method is demonstrated in Fig. 3b, which depicts representative serum from a 1-year survivor and from a non-infected control. As expected, serum from the non-infected control induced GP-specific zero to null IL-2 secretion from all four reporters. Serum from 1-year survivors induced a very marginal IL-2 GP-specific response from the FcγRIIA and FcγRIIB reporters; GP-specific IL-2 secretion from the FcγRIIIA reporter reached a level of 40 pgs/ml. In contrast, GP-specific stimulation of the FcγRI by sera from the 1-year survivor reached a level of 1400 pgs/ml. Note that differences could not be attributed to a prominent difference in the overall sensitivity of the reporters since (i) mAb-based activation of all four reporters induced similar IL-2 levels (Fig. 1b); and (ii) non-antigen specific activation by sera IgG of FcγRIIIA and FcγRI reporters induced similar IL-2 intensities (Fig. 2).

**Figure 4:**
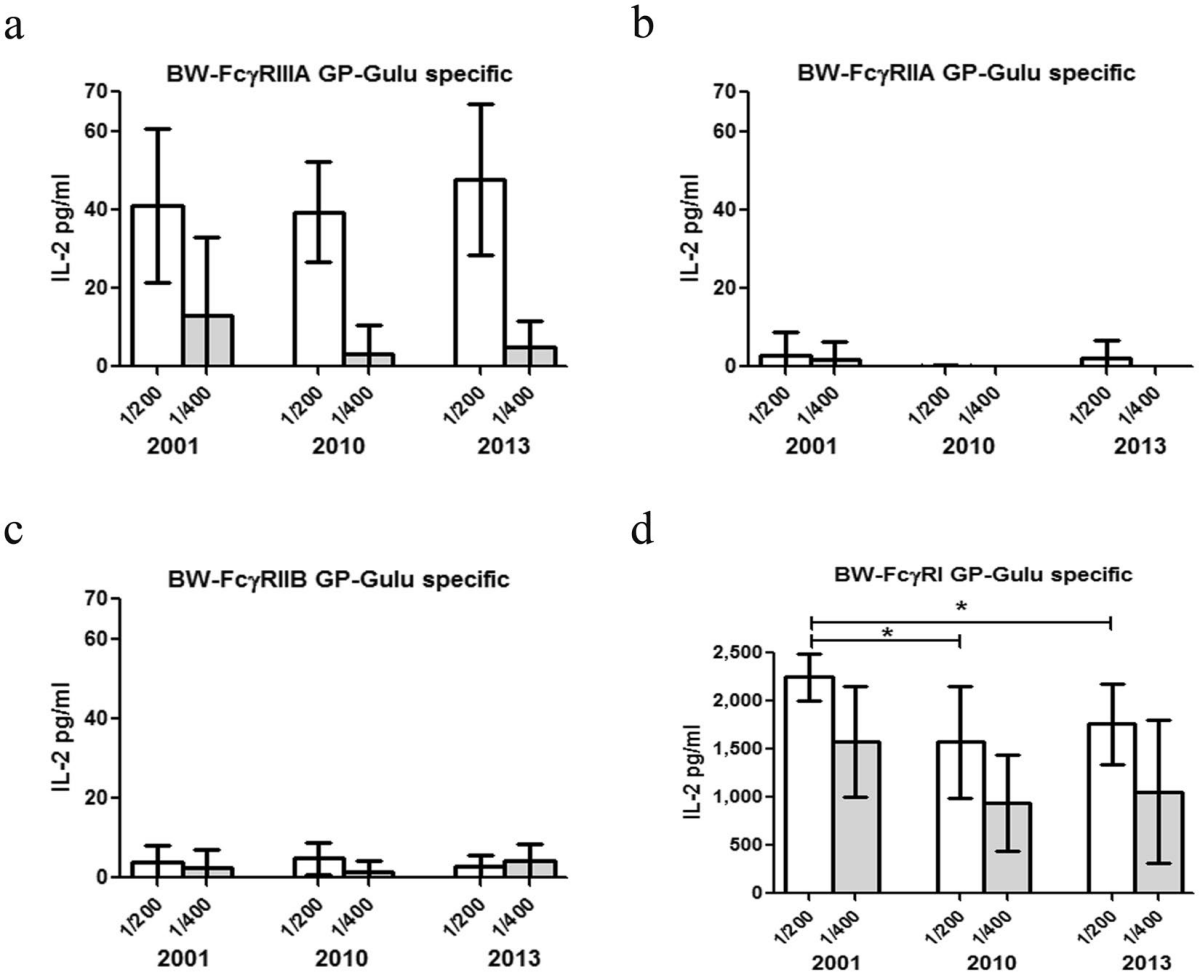
GP-specific reporters’ responses to SUDV survivors’ sera from longitudinal studies. 96 well culture plates were pre-coated with GP-Gulu antigen (5 µg/ml) and the activation assay was performed using sera samples from SUDV survivors collected during the period of 2001–2013. The sera samples were diluted 1:200 and 1:400 for all BW-FcγR cells. Supernatant was collected after 16 h and analyzed for mIL-2 by ELISA. n(2001/2013) = 7, n(2010) = 6. Two-tailed Mann Whitney test, *P value < 0.05; P value (2001–2010) = 0.0221, P value (2001–2013) = 0.0111.

The prominent differences in GP-specific stimulation of the 4 reporters shown for the 1-year survivor, motivated us to follow specific survivors over a period of 10 years from the acute ebolavirus infection. Figure 4 summarizes the GP-specific reporter responses to sera obtained from seven Ebola survivors one, ten and thirteen years post infection (2001, 2010 and 2013, respectively). The lack of GP-specific stimulation of the FcγRIIA and FcγRIIB reporters was observed for sera from all 7 survivors and at all time points tested (Fig. 4). GP-specific stimulation of FcγRIIIA reporter was low but consistent over the years. GP-specific stimulation of the FcγRI reporter by survivors’ sera was substantially higher 1 year post infection, and although with a slight reduction remained very high even 13 years post infection.

### GP-specific IgG1 is the seroprevalent subclass retained in long recovered SUDV survivors, correlating with FcγRI response

**Figure 5:**
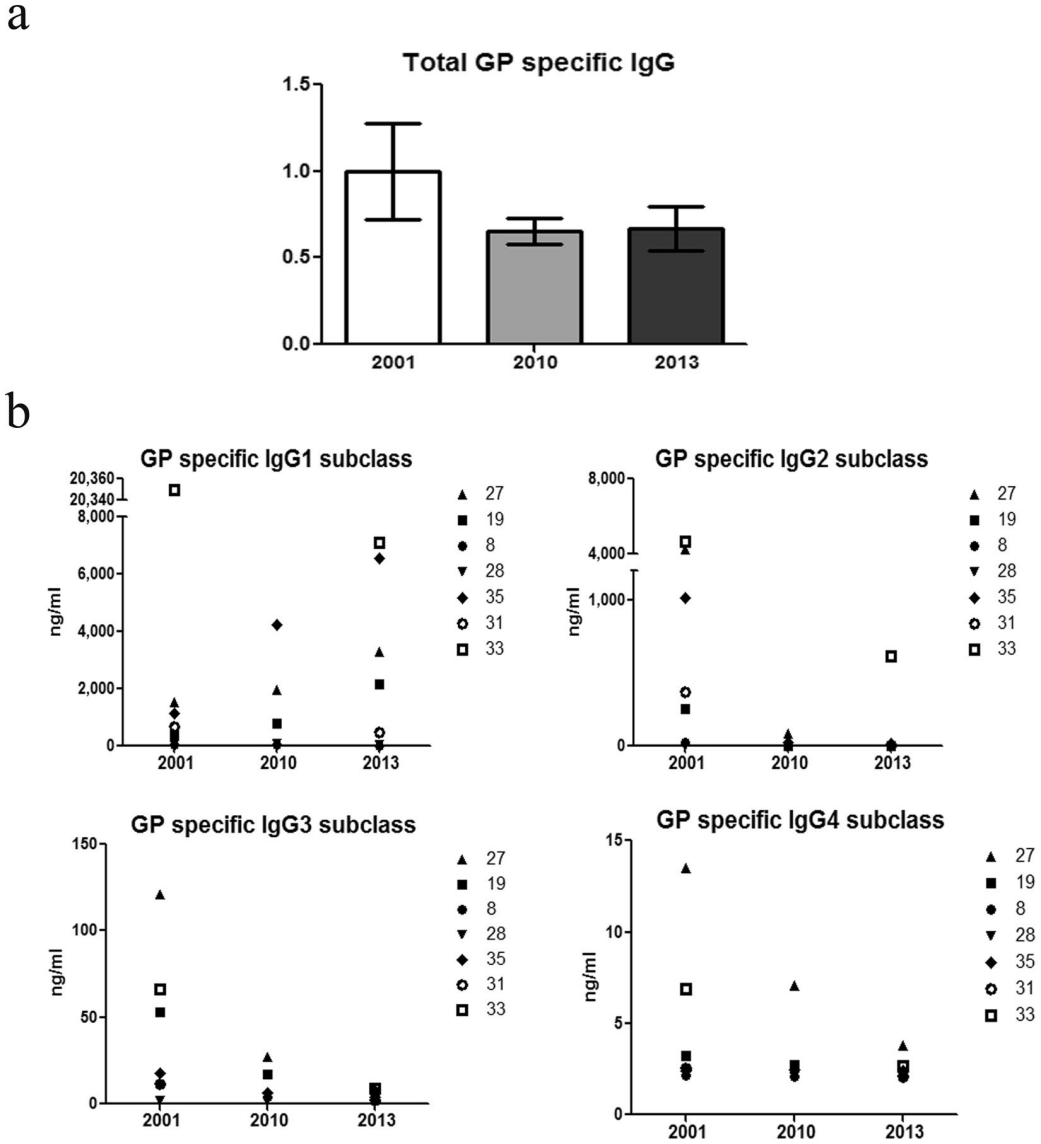
Analysis of total GP-specific IgG, and IgG subclasses in sera from SUDV survivors. To test total levels of GP-specific IgG (**a**) and IgG subclasses (**b**) in sera samples of SUDV survivors from the longitudinal study (2001-2010-2013), plates were pre-coated with GP-Gulu antigen (5 μg/ml), washed, blocked and seeded with sera samples (triplicates) diluted 1:100 for subclass analysis and 1:400 for total IgG analysis. Total levels of GP-specific IgG from 2010 and 2013 normalized to the level of GP-specific IgG from 2001.

To complement the reporter-derived GP-specific responses, we analyzed by ELISA the titer of GP-specific total IgG and IgG subclasses at these 3 time points following disease. The slight reduction between 2001 to 2010 & 2013 observed for the response of the FcγRI reporter (~25%) was also observed for total IgG binding to GP-coated wells (Fig. 5a). However, when we tested the GP-specific binding of different IgG subclasses, we observed different binding phenotypes. GP-specific IgG4 subclass binding was none to negligible in 2001 and remained none in 2010 and 2013 (Fig. 5b). Three of 7 survivors demonstrated low to moderate levels of GP-specific IgG3 subclass binding in their 2001 sera, which was reduced to low to background levels in sera from 2010 and 2013. Five of the 7 survivors’ moderate to high levels of GP-specific IgG2 subclass binding in their 2001 sera was reduced (except one) to background levels in 2010 and 2013. In contrast, GP-specific response of the IgG1 subclass demonstrated the opposite observation. Three of the 7 survivors demonstrated substantial enhancement of their GP-specific IgG1 binding over the years. One exceptionally high responder (survivor 33) demonstrated high responses for all subclasses in its 2001 sera, and these responses were reduced but still the highest in 2013 for the IgG1 and the IgG2 subclasses (Fig. 5b).

**Figure 6:**
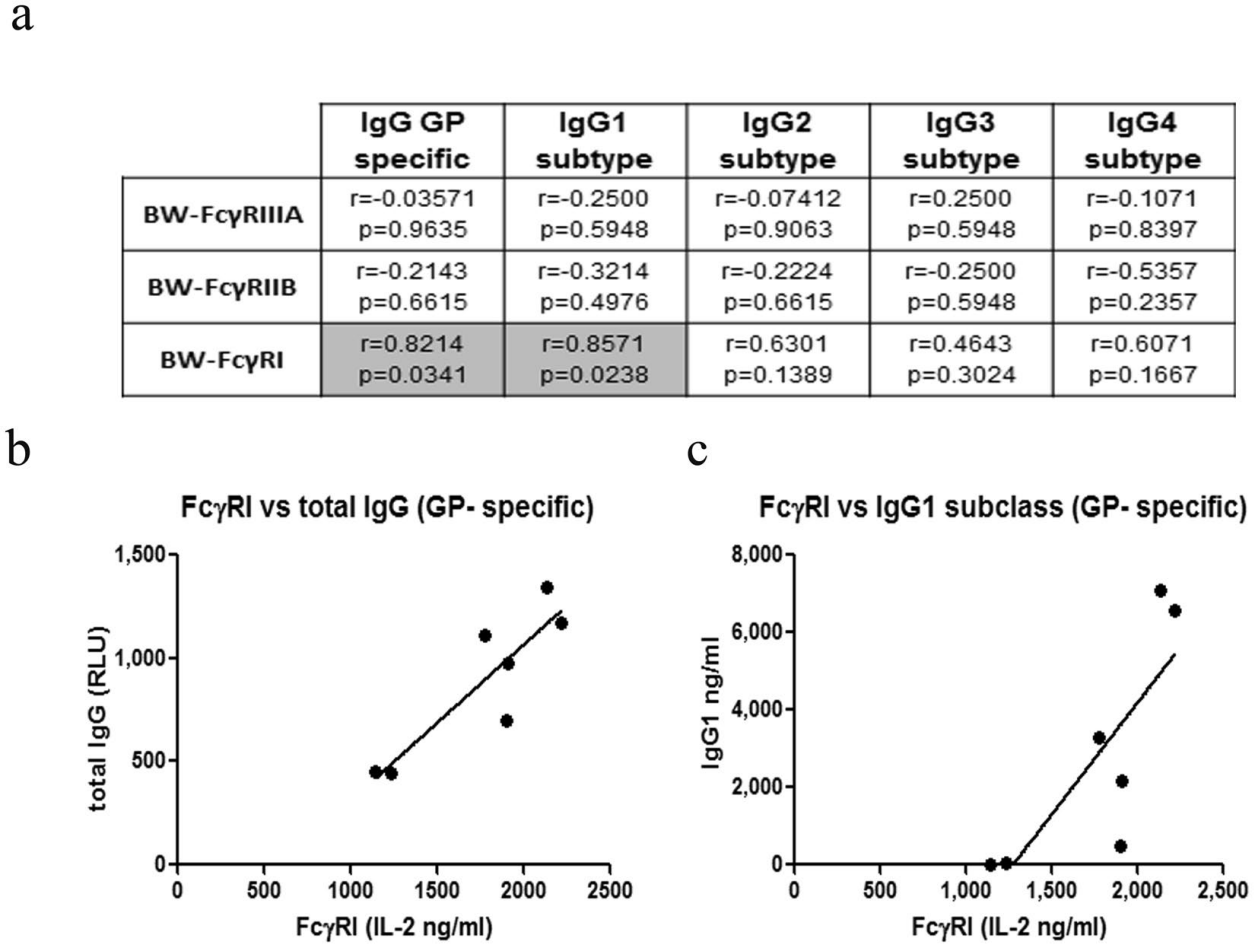
Correlation of reporters’ response and IgG levels. (**a**) Spearman rank-order correlation coefficients table for associations between reporters’ response and IgG subclasses is depicted in the 15 cells representing the tabulations. Each cell contains the corresponding spearman r-value and the probability of the association. The two grey-shaded cells have statistically significant associations, whereas the 13 clear cells have non-significant associations. (**b**,**c**) Spearman regression analysis for FcγRI and total IgG (**b**)/IgG1 subclass (**c**).

Finally, we next determined whether the different parameters that we measured for the 7 survivors correlate. We employed the Spearman Correlation assay to analyze the associations of GP-specific responses of 3 reporters (FcγRIIIA, FcγRIIB & FcγRI) and the GP-specific levels of total IgG and 4 IgG subclasses (IgG1, 2, 3 & 4). We did not analyze associations for the FcγRIIA reporter since most GP-specific results of this reporter had the zero value. Associations were studied for the 7 SUDV survivors, of which we had sera from 2001, 2010 and 2013. Of 15 cross-tabulations in the Spearman rank-order correlation coefficients table (Fig. 6a for sera from 2013), only two were significantly associated at the level of p ≤ 0.05. These two significant associations were between (i) GP-specific FcγRI reporter and GP-specific total IgG (spearman r value = 0.8214, Fig. 6b), and (ii) GP-specific FcγRI reporter and GP-specific total IgG1 subclass (spearman r value = 0.8571, Fig. 6c). Similar significance and association results were obtained when we tested the sera from 2010 (r = 0.9429 and r = 0.9429, respectively, Fig. 6). Interestingly, no significant associations were observed for the 2001 serum samples that were taken within the first year post acute infection.

## Discussion

In this study, we completed a longitudinal analysis of sera from long-recovered survivors of SUDV infection for the presence of Ebola-GP specific IgG subclasses, and for binding to Fcγ receptors. Our analysis indicated that IgG1 is the dominant GP-specific IgG subclass that remains significantly noticeable more than a decade post infection. We further demonstrated that GP-specific Abs within sera manifest prominent binding to FcγRI, as compared to binding to FcγRIIIA, FcγRIIA and FcγRIIB. To investigate the binding of IgG (Ag-specific or non-specific) to Fcγ receptors, we developed a reporter system based on the expression of CD3zeta-fused FcγRs in BW cells, which upon activation of the receptor secrete murine IL-2. The use of BW cells expressing zeta-fused Fcγ receptor for studying receptor functions was previously described by our laboratory and others (29), (31), (36). Recently, Corrales-Aguilar E *et al*. published a similar BW-based system and Promega introduced a Jurkat-based FcγR reporter system^25, 32, 33^. These published reporters include the FcγRIIIA, FcγRIIA and FcγRI and we developed similar reporters with the addition of the inhibitory FcγR, FcγRIIB. Likewise, we demonstrated that these reporters facilitate reproducible and less variable quantitative measurements of FcγR function.

Binding to FcγR, which elicits its function, represents IgG-effector functions. Two Ab-Fc modifications mediate its binding to FcγR and thus derived functionality: changes in antibody subclass and changes in a single N-linked glycan located in the CH2 domain of the IgG Fc^34^. In this regard, we found that GP-specific IgG1 is by far the seroprevalent subclass that retained and even enhanced its presence in the sera, over ten years post infection; the prevalence of other GP-specific IgG subclasses was considerably reduced over time. The unanimity of IgG1 seroprevalence results within all tested survivors is in contrast to results reported for HCMV, in which both HCMV-specific IgG1 and IgG3 retained sera activity over the years and with significant variability among individuals^35^. This variability was attributed to recurrence rate of HCMV, in which re-infections of HCMV resulted in elevation of HCMV-specific IgG3; on the other hand, persistence of IgG1 in sera was ascribed to no-recurrence of the HCMV. Therefore, GP-specific IgG1 seroprevalence in long-time survivors of SUDV could be a function of non-recurrence of the virus as reported for these survivors^12^. The recent outbreak of the Makona-type EBOV, is followed by reports of recurrence of the virus in survivors and prolonged periods of virus secretion from immune-privileged sites^36^. Hence, studying the GP-specific IgG1 and IgG3 in long-recovered Makona survivors could reveal a different phenotype as compared to the SUDV survivors.

For both HCMV and HIV-1, it was reported that pathogen-specific IgG3 Abs have greater neutralizing potential than IgG1^37, 38^. In accordance, IgG3 was secreted from a significant number of GP-specific hybridomas, generated from memory B cells of SUDV survivors secreting neutralizing mAbs (Leslie Lobel, unpublished results). We recently published that SUDV survivors, with no clinically different history, can be divided into groups with SUDV-specific neutralizing Abs, SUDV-specific non-neutralizing Abs, and with no SUDV-specific Abs^10^. Hence, the seroprevalence of GP-specific IgG1 that possibly lacks GP-mediated neutralizing activity is not associated with different clinical manifestations within the survivors.

In summary, we investigated GP-specific IgG responses in SUDV infected survivors, and demonstrated that over a decade post-acute disease, GP-specific IgG responses are significant primarily for the IgG1 subclass, and that FcγRI is the prominent FcγR associated with binding of the GP-specific IgGs in these survivors, from acute-infection to more than a decade post infection. Based on these results, Ebolavirus vaccine development should be informed by, and further investigated, with respect to the induced GP-specific IgG1 subclass and binding potency to FcγRI.

## Materials and Methods

### Cell lines

Mouse BW5147 thymoma cells (ATCC TIB-47™) and FcγR transfectants thereof were maintained in RPMI 1640 medium containing 10% (v/v) FCS, penicillin, streptomycin, glutamine, and sodium pyruvate (1 mM). BW-derived reporter cell lines were maintained in RPMI medium containing selection antibiotics.

### Human sera samples

Serum samples from infected and non-infected patients were obtained from a banked collection, as well as from surviving patients (in 2000, 2003, and 2010), in collaboration with the Uganda Virus Research Institute (Entebbe, Uganda), and shipped to Ben-Gurion University, Israel. Sera from negative controls were collected from individuals who were from the districts where the outbreak occurred but were never diagnosed with ebolavirus infection and/or were negative by polymerase chain reaction(PCR) for SUDV^12^. All serum samples used in our study were gamma irradiated and obtained under the appropriate informed consent.

### Ethics statement

The study was approved by the Helsinki committees of the Uganda Virus Research Institute in Entebbe, Uganda (reference number GC/127/13/01/15), Soroka Hospital, Beer-Sheva, Israel (protocol number 0263-13-SOR) and the Ugandan National Council for Science and Technology (UNCST) (registration number HS1332). A written informed consent was translated for and signed by each subject; a personal health questionnaire was completed for each subject at the time of blood collection. We confirm that all experiments were performed in accordance with relevant guidelines and regulations.

### Sample collection

Whole blood samples were obtained following antecubital venipuncture. Samples were directly aspirated into sterile glass vacuum tubes containing freeze-dried sodium heparin (final heparin concentration 14.3 units/ml, Becton Dickinson, Franklin Lakes, NJ, USA) and kept at 4 °C until further analysis.

### Production and selection of functional BW-reporter cell lines

Cloned sequences encoding the human extracellular portion of FcγRs fused to murine CD3ζ chain were ordered from (Invitrogen, CA USA). FcγRs included FcγRIIIA (higher affinity variant with V158^39^), FcγRIIA, FcγRIIB and FcγRI. Extracellular portions of FcγRIIA cDNA (NM_001136219.1; (34Q-217G)), FcγRIIB cDNA (NM_004001.4; (43Q-223 G) and FcγRI cDNA (NM_000566.3; (16Q-92H)) were fused to mouse CD3ζ chain cDNA (NM_001113391.2; (31L-164R)) making FcγRIIA - ζ, FcγRIIB- ζ and FcγRI- ζ respectively. The FcγR-ζ sequences were cloned into pHAGE2^40^. HEK-293T cells were transiently transfected with plasmids of interest and lentiviral packaging plasmids, and retrovirus (RV)-containing supernatants were harvested, aliquoted and employed for transduction of BW5147 thymoma cells. BW-FcγRIIIA transfectants have been previously described^41^. Following selection with Puromycin (Invivogen, CA USA) /G418 (Sigma-Aldrich, Israel), stable transfectants were screened by flow cytometry using clone 3G8 mouse anti-human CD16, clone FUN-2 mouse anti-human CD32 and clone 10.1 mouse anti-human CD64 (Biolegend, CA USA) using secondary goat anti-mouse IgG APC (Jackson immunoresearch, PA USA). BW5147 expressing receptor fused to murine CD3ζ secrete IL-2 following activation of the receptor^41^. To further test function of transfectants, cells were incubated with purified human IgGs, followed by testing with a murine IL-2 commercial sandwich ELISA kit (Biolegend, CA USA).

### Ebolavirus Sudan (Gulu) GP antigen and activation of the BW-FcγR reporter cells by IgG

Evaluation of IgG dependent activation of the BW-FcγR cells was prepared by incubating the cell lines with human sera and analyzing the supernatant from activated cells for mIL-2 by ELISA assay. 96 well cell culture plates were pre-coated with goat-Fab_2_ anti human IgG Fab_2_ 2.5 µg/ml (Jackson immunoresearch, PA USA) or Ebola GP-Gulu antigen 2.5 µg/ml^42^ diluted in 1xPBS and incubated for 2 h, blocked with 1% BSA (Sigma-Aldrich, USA) for 1 h. Human diluted samples sera (1:1 K, 1:5 K and 1:25 K; 1:200 and 1:400 dilutions respectively) were added and incubated for 1 h. BW-FcγR cells were supplemented, 50*10^3^ cells/well and incubated in RPMI 10% (v/v) FCS medium for 16–18 h. Each step was followed by a primary wash step with 1xPBS at 200 µl/well. All incubations were at 37 °C in an atmosphere of 5% CO_2_.

Ebolavirus Sudan (Gulu) GP antigen (Ebola GP-Gulu) consists of residues 1-649 of the glycoprotein, which comprises the complete ectodomain. It was expressed in HEK293T cells as a secreted protein with a HIS tag and purified over nickel chromatography by the protein core laboratory of the Nation Cancer Institute, NIH.

### Evaluation of BW-FcγR activation by mIL-2 measurement

Supernatant of activated cells (as described above) was collected after 16–18 h incubation and analyzed for mIL-2 by ELISA assay. 96 well ELISA plates were pre-coated with purified anti-mouse IL-2 (Biolegend, CA USA) using coating buffer (0.1 M, Na2HPO; pH9.0), blocked with 10% FBS in PBST (0.05% Tween-20), coated with collected supernatant followed by addition of biotinylated anti-mouse IL-2 (Biolegend, CA USA), and then SA-HRP (Jackson immunoresearch, PA USA) and TMB (Dako, Denmark) for detection of mIL-2.

### Total antigen specific IgG and antigen specific IgG subclass

To estimate Ebola antigen specific IgG total and subclass dependent activation profile: 96 well U shape plates were pre-coated with Ebola GP-Gulu antigen, blocked with 10% skim milk and human sera added (1:400 and 1:100 sera dilution respectively) followed by anti-human IgG 1–4 (Southern Biotech, Al USA) for IgG subclass, and then anti-human HRP (Jackson immunoresearch, PA USA) and peroxidase solution with luminol enhancer solution, respectively (SuperSignal West Pico, USA). Relative light units (RLU) acquired by Infinite F200 pro Elisa reader (Tecan, Austria).
